# The Coal Tar Derivatives*Read before the Atlanta Society of Medicine, February 19, 1895.

**Published:** 1895-03

**Authors:** J. W. Duncan

**Affiliations:** Atlanta, Ga.


### THE COAL-TAR DERIVATIVES.*

BY J. W. DUNCAN, M. D., ATLANTA, GA.

The coal-tar derivatives, and their combinations, are so numerous that the mere naming of them all would require as much time as I would be expected to consume in a short introductory paper this evening.

I will not attempt to give a complete list of them. Some of them are somewhat similar in their chemical composition, and quite so in their physical and therapeutical properties; while others are quite dissimilar. They are solids, semi-solids, and liquids.

*Antipyrin* and *Antifebrin*, I class as twin sisters, as I commenced their use about the same time, giving them in doses, as directed by the literature on them at that time, and I soon discovered that if I continued to use them, in heroic doses, I would kill some of my patients, so I reduced the dose to a minimum one, and got good results, without the cyanosis, and other alarming symptoms. I seldom give either one now, without combining with it some agent to modify its effects.

*Sulphonal* is an excellent hypnotic, but it should be used with discretion, and should not be continued too long, for fear of producing sulphonism.

*Tolypyrin* seems, by the history given by P. Guttman, to have an effect very much like antipyrin.

*Trional*, I have used only in one case. Did not get any results in the ordinary dose.

*Hypnal*, a combination of chloral and antipyrin, is recommended in chorea, delirium tremens, and insomnia. I have not tried it.

*Phediuretin* acts as a diuretic, probably by its effect on the central nervous system.

*Phenacetin* is diaphoretic, antipyretic, analgesic, and antiseptic, and may be used locally, as well as constitutionally. It is one of the leading members of this large family in my practice.

*Phenocoll Hydrochlorate* is said to be antipyretic, analgesic, antiperiodic, etc.

*Read before the Atlanta Society of Medicine, February 19, 1895.

*Salacetol*, introduced by P. Fritsch, is intended as a substitute for salol, and, if it be better than salol, it is a good one.

*Salipyrin* is a salicylate of antipyrin, and has nothing to specially recommend it.

*Salocol*, according to P. Schering, has several good features. He says it is antipyretic, antineuralgic, and antirheumatic, certain in its action, and presenting little danger. The dose is large—from fifteen to thirty grains, several times a day.

*Salol.*—To my mind, this is one of the most useful of the list, being antiseptic, analgesic, antirheumatic, antipyretic, antifermentative, etc. I have used it extensively in diarrhœa, dysentery, rheumatism, typhoid fever, intestinal fermentation, tympanitis, etc.

*Salophen*, Koch says, acts promptly in rheumatism, and he thinks it is better than salicylate of soda, or salol. The dose is large—one and a half drachms, daily.

*Steresol* is a new varnish-like substance, used for dressing sores, etc.

*Formanilid* is the homologue below acetanilid. It is claimed that it will cause complete anesthesia, and that it is more efficacious than cocaine, the anesthesia lasting longer.

*Aniline*—methylene blue—is used internally and hypodermically, in doses of one-half to seven grains, two or three times a day, in rheumatism and neuralgia, and to reduce temperature.

*Analgene* is used for the same purposes as antipyrin. It is tasteless, but causes the urine to be red, which may alarm the patient.

*Antinervine* is a new remedy for colds and rheumatism.

*Antipyrin* has been in use for some years, and is recognized as an antipyretic, antiseptic, analgesic, etc.

*Asaprol* is said to be especially used in rheumatism. It is said to be preferable to salol, or salicylate of soda, as it does not cause tinnitus aurium, and it is not contra-indicated in albuminuria.

*Beta-Naphthol*, from which the above is derived, is highly-praised for its antiseptic properties, in gastric and intestinal affections. I have used it only in one case, and that was a case of consumption. I used it hypodermatically. It seemed to control the fever. No bad effects followed, except some soreness at point of injection.

*Benzosol* is highly-praised in diabetes mellitus.

*Bismuth-naphthol-hydrated* is said to be a nontoxic antiseptic, and will arrest fermentation at once. Claimed to be useful in intestinal disorders, etc.

*Bismuth-tribromphenol* is a new antiseptic, and is one of the late specifics against cholera.

*Bismuth-Beta-Naphthol* is praised for its effects in diarrhœa.

There are several other combinations of the coal-tar derivatives, with bismuth-phenates, ete.

*Carbolic Acid* is one that has been in general use for many years, and is still a favorite with us all. You are all familiar with its uses and virtues.

*Exalgin* relieves pain, and is said to be useful in chorea.

And what more shall I say, for time would fail me to speak of sulphaminol, tetrinol, antikamnia, monobrom-phenol, naphtalin, phenyl-hydrazin, phenosalyl, resorsin, thermodin, antitoxine, salphen, tolerine, trecosal, trecresalimine, picric acid, saccharine, anthrarobin, asaprol, benzo guiacol, betol, creosote, creolin, cresolol, gallanol, gallabromol, guiacol, hydroquione, latrol, ichthyol, lysol, salicylic acid, phenylacetic, amido phenol, alumnol, sulpho-salicylic, etc.

I will close by stating some conclusions, from my clinical experience with the coal-tar derivatives. They are valuable additions to our therapeutics. They are almost or quite indispensable to the surgeon or gynecologist, as well as to the general practitioner. When judiciously used, in appropriate cases, the results are good; but when given in heroic doses, and continued for a long time, they are as potent for evil.

The more active antipyretics of this class should not be relied upon to control hyperpyrexia, in the continued fevers, or in pneumonias, as we have other remedies, which are equally as efficacious, and which are not so likely to produce injurious blood changes.

I have often observed the slow convalescence of patients recovering from fevers, after the use of coal-tar derivatives, and I have reason to believe that relapses are more common, also.

In the ephemeral fevers, catarrhal fever, neuralgias, rheumatism, la grippe, etc. (when not complicated with pneumonia), I have been very well satisfied with their effects. As a rule, the dose need not be large, and should not be continued for too long a time.

I have used creolin quite frequently, and believe it to be an excellent antiseptic. I have tried it diluted, as an anema, in dysentery, but was not satisfied with its effects. It caused too much pain, and I failed to observe any curative action.

I have not used, in my practice, all the agents mentioned in this paper, and I doubt if I ever do, and I have only mentioned the names of a few of this prolific family. But I hope that I have said enough to elicit a full and free discussion.

---

### A GEM, DEDICATED TO THAT RABELAIS OF THE PROFESSION, T. C. M.

(*Cincinnati Lancet-Clinic.*)

During the last two years our old war pensioners have been put to serious inconvenience, and great loss in some cases, by the exactions of the indefatigable Hoke Smith, and I want to relate an incident in connection with a case of this kind.

Captain K——— was threatened with a 50 per cent. reduction of his pension, and was ordered before the pension board to sustain the existence of his long-continued disabilities. Then he was ordered to undergo examination by a civil surgeon, then back to the board again. Then he was asked to get some general evidence from his family physician, and also to get the affidavits of two or three other physicians to substantiate the fact that he had an inguinal hernia, in connection with a chronic diarrhœa. The Captain listened to the learned dissertations of the surgeons in their differential diagnosis of hydrocele and hernia until he got bewildered, and finally penned Hoke Smith the following epistle:

DEAR MR. SMITH—I have been sent around from pillar to post, from poor doctors to worse ones, and I want to tell you a story. One beautiful summer evening a couple of young lovers might have been seen lounging in close proximity to each other, beneath a budding apple tree, tenderly caressing each other. The young lady was clad in those clinging, expensive robes, which betokened that she was the child of rich, but respectable parents, and the young man also had the earmarks of prosperity. Suddenly from out the stillness came the soft, sweet voice of the maiden, who said: "Algernon, I discover that your right kidney hangs a little lower than the left, doesn't it?" Mr. Smith, please send me to some doctor who knows the difference between a kidney and a testicle.

